# Globus Pallidus Internus Deep Brain Stimulation for Disabling Diabetic Hemiballism/Hemichorea

**DOI:** 10.1155/2017/2165905

**Published:** 2017-10-23

**Authors:** Byung-chul Son, Jin-gyu Choi, Hak-cheol Ko

**Affiliations:** ^1^Department of Neurosurgery, Seoul St. Mary's Hospital, College of Medicine, The Catholic University of Korea, Seoul, Republic of Korea; ^2^Catholic Neuroscience Institute, College of Medicine, The Catholic University of Korea, Seoul, Republic of Korea

## Abstract

Unilateral hemichorea/hemiballism (HH) associated with contralateral neuroimaging abnormalities of the basal ganglia, which is characterized by T1 hyperintensity on magnetic resonance imaging (MRI) and is secondary to diabetic nonketotic hyperglycemia, is a rare and unique complication of poorly controlled diabetes mellitus (DM). Although almost all prior reports have documented rapid resolution of HH within days after normalization of blood glucose levels, medically refractory persistent HH has been noted. The experience of surgical intervention for persistent HH is limited. A 46-year-old, right-handed female patient with type 2 DM presented with refractory diabetic HH on the left side of 6 months' duration despite DM control and neuroleptic medication usage. Image-guided deep brain stimulation (DBS) on the right globus pallidus internus (GPi) was performed. A mechanical micropallidotomy effect was observed and chronic stimulation of GPi was quite effective in symptomatic control of diabetic HH until a 16-month follow-up visit. DBS of the GPi can be an effective treatment for medically refractory diabetic HH.

## 1. Introduction

Hemichorea/hemiballism (HH) is a hyperkinetic movement disorder characterized by acute or subacute onset of high-amplitude, involuntary movements affecting one side of the body [1, 2]. Ischemic/hemorrhagic stroke within the contralateral subthalamic nucleus (STN) and basal ganglia and nonketotic hyperglycemia are the most common causes of HH. Diabetic HH is a unique syndrome characterized by HH, hyperglycemia, and striatal hyperintensity on T1-weighted magnetic resonance image (MRI) [3].

The clinical course of diabetic HH is favorable and the symptoms tend to resolve rapidly with normalization of hyperglycemia in patients who develop diabetic HH secondary to nonketotic hyperglycemia [2, 4]. However, there are rare patients who continue to have persistent HH years after the inciting hyperglycemic crisis [5]. In rare, medically refractory diabetic HH, stereotactic lesioning of the ventrolateral (VL) thalamus [6] and GPi [7] and DBS of the thalamic ventralis oralis (VO) nucleus [8] have been reported to show significant benefit in case reports. Although GPi DBS was reported to be effective in a patient with HH secondary to vascular insult to the STN and substantia nigra [9], its effectiveness in diabetic HH has not yet been reported. We report the effectiveness of chronic GPi stimulation in a patient suffering refractory diabetic HH and summarized the utility of stereotactic surgery in this rare hyperkinetic movement disorder of metabolic cause.

## 2. Case Presentation

A 46-year-old, right-handed female patient with an 11-year history of type 2 diabetes mellitus (DM) presented with a continuous, violent choreic/ballistic movement of the left arm, leg, and trunk of 6 months' duration. At the beginning, she experienced vague discomfort in her left arm after a brief episode of heavy lifting. An involuntary choreic movement gradually developed in her arm within several hours. Owing to cramping pain associated with a flinging movement in her left arm and hand, she was initially treated with nonsteroidal anti-inflammatory drugs and physical therapy. However, the choreic movement progressively worsened over the following 2 weeks and eventually became ballistic and involved her left arm and leg and had a high amplitude. She was admitted to another hospital via the emergency department for evaluation. Neurologic examination showed facial dyskinesis (grimacing) and a ballistic movement in her left trunk, arm, and legs. The movements could not be suppressed voluntarily but ceased during sleep. Muscle tone and strength in the upper and lower extremities were normal bilaterally. There was no sensory impairment and her cranial nerves were normal. Laboratory studies revealed a fasting blood glucose level of 536 mg/dl, a serum osmolarity of 335 mOsm/kg, and an HbA1c count of 15.7%. Urinalysis was negative for ketones. There was no history of dopamine agonist or estrogen medication use or rheumatic fever/Sydenham's chorea.

**Figure 1:**
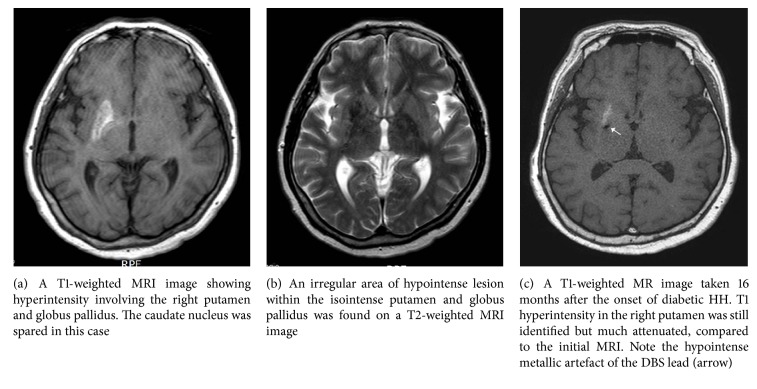
Magnetic resonance imaging (MRI) findings of diabetic hemichorea/hemiballism.

A T1-weighted MRI image revealed a region of increased signal intensity restricted to the right putamen and globus pallidus, which was isointense on T2-weighted images (Figure 1). No abnormal enhancement with gadolinium was found. Under a diagnosis of nonketotic hyperglycemic hyperosmolar syndrome, the patient's blood glucose level was controlled with subcutaneous injection of insulin. The severe ballistic movement progressively improved with titration of haloperidol (up to 10 mg/day), clonazepam (6 mg/day), amantadine (300 mg/day), and tiapride (200 mg/day) for HH. Clinical manifestations progressively improved over the course of 3 months of DM control with neuroleptics but did not disappear. She suffered continuous HH, which was most severe in the left leg and foot and involved a severe gait impairment. Finally the patient was referred to our clinic for surgical treatment for chronic, disabling HH at 6 months after the onset of diabetic HH. Considering the chronicity and medical intractability of the situation, GPi DBS was decided upon.

**Figure 2:**
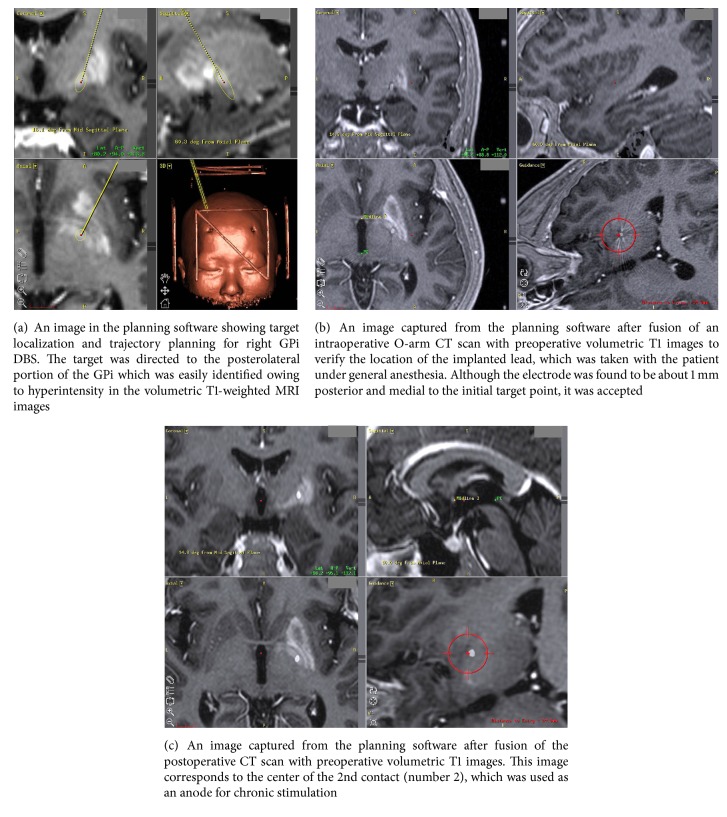
Image-guided deep brain stimulation (DBS) of the globus pallidus internus (GPi) for refractory diabetic HH.

The patient underwent a stereotactic MRI scan (1.5 tesla Archieva®, Philips, Best, The Netherlands), and volumetric T1-weighted three-dimensional images, T2-weighted images, and proton density images were obtained. The images were transferred to a Framelink® planning station (version 4.1, Medtronic, Minneapolis, MN, USA) so that we could determine the coordinates of the image-guided GPi target and were reformatted for extraventricular trajectory planning (Figure 2(a)) [10, 11]. Implantation of the electrode (model 6149, St. Jude Medical, Plano, Texas, USA) was performed under C-arm fluoroscopy guidance under the general anesthesia. After implantation of the lead, “O-arm” intraoperative CT scan (Medtronic, Minneapolis, MN, USA) was performed and the images were again transferred to the Framelink® planning station and merged with the preoperative volumetric T1-weighted MR images to verify the location of the electrodes (Figure 2(b)). After verification of electrode location, the distal end of the electrode was tunneled subcutaneously for external stimulation. After the patient recovered from anesthesia, an immediate postoperative computed tomography (CT, 1 mm thick axial slices) scan was taken again for verification of the location of the lead with the preoperative T1 MR images and intraoperative O-arm images (Figure 2(c)).

A prominent mechanical effect with significant reduction (50% reduction in intensity) of the choreic and proximal ballistic movements was observed. Bipolar electrical stimulation (100–130 Hz, 1–3 mA, 130 usec, up to 3.5 mA) further suppressed HH, and the hyperkinetic movements became grossly undetectable after 7 days of external stimulation. No stimulation-related side effects such as dysarthria, dysphasia, or motor contraction were observed. A pulse generator (Libra®, St. Jude Medical, Plano, TX, USA) was implanted via a transaxillary subpectoral route after 7 days of external stimulation [12]. The patient returned to her usual activities and became reemployed in her previous job on diabetic medication control and clonazepam (1.5 mg/day). However, she felt continuous cramping pain and an internal sensation of muscular contraction in her lower leg and foot without any grossly visible choreic movements when walking. Chronic GPi stimulation was performed with regular adjustment of the stimulation parameters (2.6–3.0 mA, 110–130 Hz, 110–120 usec, 1 (−), 2 (+)) every 3 months. At a 16-month postoperative follow-up, the patient was grossly free of HH. However, minimal chorea in the left calf and foot was noted. She was readmitted for an on and off stimulation study and a follow-up MRI examination. After 30-minute off stimulation, the ballistic movements of the left thigh, distal leg, and shoulder returned and gait difficulty was noticed along with chorea in the left foot. After 10 minutes of on stimulation, the HH in her left leg and foot disappeared almost completely. Her fasting blood glucose, serum osmolarity, and HbA1c level were 109 mg/dl, 291 mOsm/kg, and 7.9%, respectively. T1-weighted MRI images again revealed a high signal intensity confined to the right putamen, which was greatly attenuated compared with that seen on the initial preoperative MRI scan (Figures 1(a) and 1(c)).

## 3. Discussion

### 3.1. Diabetic HH

Transient chorea/ballism provoked by an episode of nonketotic hyperglycemia has repeatedly been reported over the past couple of decades and is the second most common cause of hemiballism [12]. The blood glucose level in patients with the condition ranges from 500 to 1000 mg/dL during the hyperglycemic precipitating crisis. HH is commonly associated with type 2 DM and is rarely associated with type 1 DM. Although diabetic HH mostly occurs when patients are in a state of nonketotic hyperglycemia, some patients also have ketotic hyperglycemia [13]. The movements are typically unilateral but can be generalized in rare cases [5]. Description of the movements ranges from mild chorea to severe ballism. According to a review of 53 cases of diabetic HH [4], the average age of onset is 71, and there is a female predominance of 1.8 : 1. Genetic factors may play a role, with an Asian predominance in reported cases [4].

High intensity in the basal ganglia on T1-weighted MRI is a characteristic finding in diabetic HH [2–6, 14, 15]. T1 striatal hyperintensity involves the putamen in all cases, the head of the caudate nucleus in most cases, and the globus pallidus in a minority of cases [14, 15]. In contrast to typical T1 hyperintensity, the findings on T2-weighted images are variable and range from hyper- and iso- to hypointensity. Gradient-echo and gadolinium-enhanced T1-weighted images are reported to be normal. Several mechanisms have been proposed to explain T1 striatal hyperintensity, including acute ischemia, petechial microhemorrhage, injury secondary to hyperviscosity, and vasogenic edema [14]. Currently, hyperviscosity is suggested to be the most plausible mechanism because of the following findings [14]: elevated serum osmolarity at the time of HH, variable T2-weighted MR signal changes that reflect the difference in patterns and severity of hyperviscosity, and elevated myoinositol and choline levels seen on MR spectroscopy that are in line with a finding of abundant gemistocytes with increased protein content [14–17].

The putamen has been suggested to play a central role in diabetic HH [14]. The putamen has abundant medium-sized spiny neurons which store and release gamma amino butyric acid (GABA) [18], and there is universal involvement of the putamen in diabetic HH [14, 15]. Therefore, dysfunction of GABAergic neurons in the putamen may result in a disturbance of output nuclei of the basal ganglia, such as the substantia nigra and GPi [14]; resultant disinhibition of the motor thalamus ensues, leading to hyperkinetic movement disorder.

### 3.2. Treatment of Diabetic HH

Diabetic HH generally resolves over time, and often no treatment is necessary. Determining the etiology of the condition is a matter of paramount importance and correcting nonketotic hyperglycemia along with long-term diabetic control is the mainstay of treatment [2, 4, 12]. In cases of prolonged diabetic HH, dopamine receptor blocking agents (chlorpromazine or haloperidol), neuroleptics (olanzapine, clozapine), and dopamine depleting agents (tetrabenazine) have been reported to be beneficial [2]. However, there are some rare patients who continue to have persistent HH years after the inciting hyperglycemic crisis [5]. The exact incidence of medically refractory diabetic HH is unclear. In rare, medically refractory diabetic HH, stereotactic surgery has sporadically been reported in single case reports and we summarized them in Table 1.

Owing to the limited reports on surgical intervention against diabetic HH, the timing and type of surgery, lesioning, or DBS are difficult to determine. However, despite the limited evidence, both lesioning and stimulation in the VL or VO thalamus and GPi showed good results [6–8]. Because diabetic HH is especially prevalent in Asians, all 3 cases regarding surgical treatment came from Japan [6–8]. We chose the GPi as a target of surgical intervention because previous reports regarding GPi lesioning produced uniformly positive results against diabetic HH [7] as well as HH secondary to vascular insults involving the STN and the brain stem [9, 19–21]. In addition, determination of the target within the GPi and trajectory planning was easily done with stereotactic MR images owing to prominent T1 hyperintensity in the GP (Figure 2(a)). A case of DBS of the VO thalamic nucleus was reported to be effective in diabetic HH [8], and we also achieved significant long-term effectiveness with GPi stimulation. Owing to the rarity of diabetic HH, the present case is the first report of GPi DBS for refractory diabetic HH. The present case showed persistent choreiform dyskinesia during off GPi stimulation even after 16 months following the onset of diabetic HH despite long-term continuous diabetic control and medical treatment with much attenuation of T1 hyperintensity in the follow-up MRI. Therefore, timely surgical intervention would be of help in alleviation of disabling motor symptoms in rare patient with persistent diabetic HH.

## 4. Conclusion

We report the long-term effectiveness of GPi DBS in the treatment of chronic, disabling diabetic HH.

## Figures and Tables

**Table 1:** Summary of stereotactic surgery for diabetic hemichorea/hemiballism.

Study/year	Number of patients	Age/sex	Timing of surgery	Surgery (lesion/DBS)	Results	follow-up period	Remarks
Takamatsu et al. [6], 1995	1	57/f	1.5 mo.	VL lesion	Excellent	4 years	
Nakano et al. [8], 2005	1	65/m	5 mo.	VO (Voa, Vop) DBS	Effective	9 mo.	Persistent HH in off stimulation
Goto et al. [7], 2010	1	78/f	N/A	GPi lesion	Excellent	12 mo.	HH immediately disappeared
Current case, 2017	1	46/f	6 mo.	GPi DBS	Effective	16 mo.	Persistent HH in off stimulation

GPi: globus pallidus internus; HH: hemichorea/hemiballism; mo: months; VL: ventral lateral nucleus; VO: ventralis oralis; Voa: ventralis oralis anterior; Vop: ventralis oralis posterior.

## References

[1] Jankovic J., Fahn S., Jankovic J., Fahn S. (2007). Chorea, ballism, athetosis. *Principles and Practice of Movement Disorders*.

[2] Hawley J. S., Weiner W. J. (2012). Hemiballismus: current concepts and review. *Parkinsonism & Related Disorders*.

[3] Hashimoto T., Hanyu N., Yahikozawa H., Yanagisawa N. (1999). Persistent hemiballism with striatal hyperintensity on T1-weighted MRI in a diabetic patient: A 6-year follow-up study. *Journal of the Neurological Sciences*.

[4] Oh S.-H., Lee K.-Y., Im J.-H., Lee M.-S. (2002). Chorea associated with non-ketotic hyperglycemia and hyperintensity basal ganglia lesion on T1-weighted brain MRI studya meta-analysis of 53 cases including four present cases. *Journal of the Neurological Sciences*.

[5] Ahlskog E. J., Nishino H., Evidente V. G. H. (2001). Persistent chorea triggered by hyperglycemic crisis in diabetics. *Movement Disorders*.

[6] Takamatsu K., Ohta T., Sato S. (1995). Two diabetics with hemichorea hemiballism and striatal lesions. *No To Shinkei*.

[7] Goto T., Hashimoto T., Hirayama S., Kitazawa K. (2010). Pallidal neuronal activity in diabetic hemichorea-hemiballism. *Movement Disorders*.

[8] Nakano N., Uchiyama T., Okuda T., Kitano M., Taneda M. (2005). Successful long-term deep brain stimulation for hemichorea-hemiballism in a patient with diabetes. *Journal of Neurosurgery*.

[9] Hasegawa H., Samuel M., Jarosz J., Ashkan K. (2009). The treatment of persistent vascular hemidystonia-hemiballismus with unilateral GPi deep brain stimulation. *Movement Disorders*.

[10] Son B. C., Shon Y. M., Choi J. G. (2016). Clinical outcome of patients with deep brain stimulation of the centromedian thalamic nucleus for refractory epilepsy and location of the active contacts. *Stereotactic and Functional Neurosurgery*.

[11] Son B. C., Han S. H., Choi Y. S. (2012). Transaxillary subpectoral implantation of implantable pulse generator for deep brain stimulation. *Neuromodulation: Technology at the Neural Interface*.

[12] Postuma R. B., Lang A. E. (2003). Hemiballism: revisiting a classic disorder. *The Lancet Neurology*.

[13] Abe Y., Yamamoto T., Soeda T. (2009). Diabetic striatal disease: Clinical presentation, neuroimaging, and pathology. *Internal Medicine*.

[14] Kandiah N., Tan K., Lim C. C. T., Venketasubramanian N. (2009). Hyperglycemic choreoathetosis: Role of the putamen in pathogenesis. *Movement Disorders*.

[15] Battisti C., Forte F., Rubenni E. (2009). Two cases of hemichorea-hemiballism with nonketotic hyperglycemia: A new point of view. *Neurological Sciences*.

[16] Chu K., Kang D. W., Kim D. E., Park S. H., Roh J. K. (2003). Diffusion-weighted and gradient echo magnetic resonance findings of hemichorea-hemiballismus associated diabetic hyperglycemia: a hyperviscosity syndrome?. *Archives of Neurology*.

[17] Shan D. E., Ho D. M., Chang C., Pan H. C., Teng M. M. (1998). Hemichorea-hemiballismus: an explanation for MR signal changes. *American Journal of Neuroradiology*.

[18] Graybiel A. M. (1990). Neurotransmitters and neuromodulators in the basal ganglia. *Trends in Neurosciences*.

[19] Suarez J. I., Verhagen Merman L., Reich S. G., Dougherty P. M., Hallett M., Lenz F. A. (1997). Pallidotomy for hemiballismus: Efficacy and characteristics of neuronal activity. *Annals of Neurology*.

[20] Vitek J. L., Chockkan V., Zhang J.-Y. (1999). Neuronal activity in the basal ganglia in patients with generalized dystonia and hemiballismus. *Annals of Neurology*.

[21] Yamada K., Harada M., Goto S. (2004). Response of postapoplectic hemichorea/ballism to GPi pallidotomy: Progressive improvement resulting in complete relief. *Movement Disorders*.

